# 3D Printed Deformable Surfaces for Shape-Changing Displays

**DOI:** 10.3389/frobt.2019.00080

**Published:** 2019-08-28

**Authors:** Aluna Everitt, Jason Alexander

**Affiliations:** School of Computing and Communications, Lancaster University, Lancaster, United Kingdom

**Keywords:** shape-changing displays, actuated tangible interfaces, 3D printing surfaces, design and fabrication approach, deformable interfaces

## Abstract

We use interlinked 3D printed panels to fabricate deformable surfaces that are specifically designed for shape-changing displays. Our exploration of 3D printed deformable surfaces, as a fabrication technique for shape-changing displays, shows new and diverse forms of shape output, visualizations, and interaction capabilities. This article describes our general design and fabrication approach, the impact of varying surface design parameters, and a demonstration of possible application examples. We conclude by discussing current limitations and future directions for this work.

## Introduction

Shape-changing displays are an emerging technology enabling active shape input and output through computationally controlled actuation. The dynamic movement of the display's surface enables new forms of data representations, such as active elevated physical topography, and novel tangible interactions, such as physical sculpting, that are beyond the capabilities of conventional flat-screen 2D displays (Alexander et al., 2018). Current implementations focus mainly on pin array actuators, where each actuator represents a physical pixel in a 2D array that changes its vertical position based on input or output (Taher et al., 2017b). Conventional fabric surfaces are also used to create continuous fluid surface deformations (Sturdee and Alexander, 2018).

**Figure 1:**
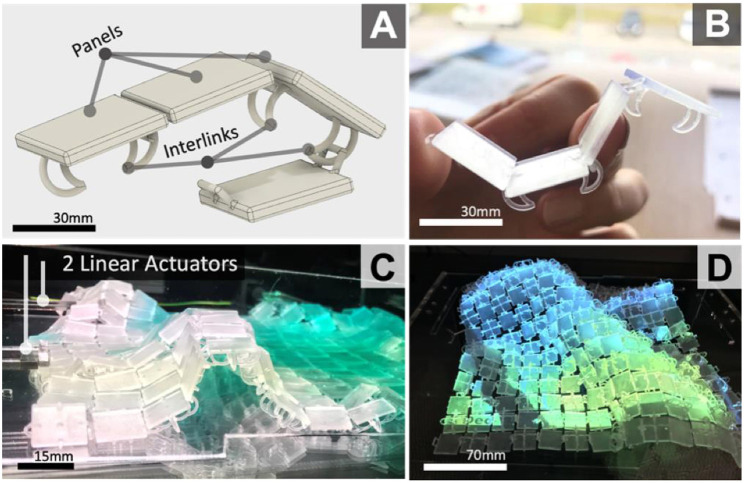
Basic 3D model **(A)** and 3D print **(B)** of interlinked panels, and fabricated shape-changing 53 displays examples **(C,D)**.

We aim to expand the design space of fabrication approaches for shape-changing displays through the exploration of interlinked 3D printed surfaces that deform using both vertical and horizontal actuation. 3D printed fabrics and textiles are becoming an emergent application area in digital fabrication (Rosenkrantz, 2016). By mimicking interlinking textile structures, we can create surfaces with the combined qualities of flexibility and rigidness for moving shape forms (Figures 1C,D). These surfaces can be adapted in scale and resolution via computer-aided design (CAD) for diverse uses, from small scale wearables to larger scale installations.

We propose a reproducible, low-cost rapid fabrication technique for shape-changing displays by enabling users to design interlinked 3D printed surfaces that can adapt in fluidity/rigidness. Our core fabrication concept is to use 3D printed panels, that are interlinked (see Figures 1A,B) during the printing process, to create deformable continuous surfaces, specifically for shape-changing displays (Figures 1C,D). Each panel is rigid, but in aggregate they behave as a continuous surface. Unlike cloths and fabrics, previously used for shape displays, these surfaces can adapt in fluidity or rigidness based on their designs. By enabling direct manipulation of surface properties, during the design stages, we believe this fabrication approach will further enhance the design and development of shape-changing displays. Using new (e.g., horizontal force) and existing actuation technologies (e.g., pin-arrays) we show how this fabrication technique can be adopted to shape-changing displays. Our Supplementary Video 1 showcases actuation and interaction capabilities of our 3D printed deformable surface and an application example of it as a shape-changing display.

First, we discuss current implementation techniques for shape-changing displays and 3D printed fabrics. We then present our general design and fabrication approach for 3D printing interlinked surfaces including the impact of varying design parameters (e.g., interlink and panel dimensions). We then demonstrate scalability and the technical opportunities these surfaces offer, such as horizontal actuation for surface deformations. Vertical actuation was also tested with a pre-existing shape-changing display (Taher et al., 2015) to demonstrate generalizability. Finally, we discuss future directions of our work, limitations, and possible applications.

In summary, our work contributes the application of 3D printed deformable surfaces as a novel approach to further the development of shape-changing displays beyond current state-of-the-art. The 3D printed interlinked surfaces we fabricate show: (1) fewer actuators needed for dynamic surface deformations, together with horizontal force actuation. (2) Opportunities for under-the-surface visualization and embedding interactive components into the surface as well as retained rigidness whilst rendering cylindrical, oval, and tunnel forms.

## Related Work

As an emerging technology, shape-changing displays offer physical change of form as input and/or output through interactivity and computational control (Rasmussen et al., 2012). Poupyrev et al. (2007) provide an overview of actuation mechanisms and techniques that combine image and dynamic shapes. Coelho and Zigelbaum (2011) survey shape-changing materials and their primary dynamic properties; while recent reviews classify (Rasmussen et al., 2012; Sturdee and Alexander, 2018) current state-of-the-art for shape-changing displays and interfaces. Commonly, shape-changing displays consist of a 2D array of motorized linear actuation pins (Poupyrev et al., 2004; Leithinger and Ishii, 2010; Follmer et al., 2013; Leithinger et al., 2013; Ishii et al., 2015; Jang et al., 2016) or deformable surface materials (Dand and Hemsley, 2013; Tsimeris et al., 2013; Yao et al., 2013; Sahoo et al., 2016). Our work builds on this previous research, specifically on shape-displays with motorized linear actuators for this initial exploration. We use commercially available actuators [e.g., ShapeClips (Hardy et al., 2015)] as opposed to other shape-changing materials discussed by Coelho and Zigelbaum (2011) that are less accessible.

### Mechanical Pin-Actuation Displays

Relief (Leithinger and Ishii, 2010) combines 120 motorized pin actuators with a Lycra layer for continuous surface deformations and project imagery from above. inForm (Follmer et al., 2013) consists of a 30 × 30 array of motorized pins. Lumen (Poupyrev et al., 2004) and EMERGE (Taher et al., 2015) embed a light source (e.g., LED) to each actuator pin, mitigating occlusion. Tilt Displays (Alexander et al., 2012) have high resolution embedded visual displays, but lower resolution physical output. Though these approaches mitigate occlusion, imagery or shape resolution is compromised. Rendering complex polygonal structures, cylindrical meshes, or curved contours is also limited due to a lack of dynamicity in surface configurations. We combine flexible and continuous surface qualities whilst reducing the number of actuators to create shape-output.

### Elastic Deformable Displays

TableHop (Sahoo et al., 2016) is an elastic self-actuated display surface, with a 3 × 3 grid of transparent electrodes. Rear-projection retains high-resolution visuals without occlusion. It achieves ±5 mm surface deformation. We also use a translucent surface to mitigate occlusion with rear-projection. However, we render greater (>5 mm) surface deformation, cylindrical and oval forms. We also reduce the number of actuators needed for surface deformations to limit costs. PolySurface (Everitt and Alexander, 2017) is a prototyping approach for shape-changing display that combines Spandex with solid laser cut segments to reduce actuation requirements. We also combine solid elements with flexibility, but as a single uniformed layer of panels interlinked during printing to reduce assembly requirements.

### Actuation Techniques From Robotics

There is an increasing interest in developing reconfigurable surfaces in the field of robotics. The cross-disciplinary contributions of this work aim to extend the utility and accessibility of tangible robotic interfaces for future applications within a range of domains. A variety of actuation techniques, that go beyond mechanical linear motorized actuators, have been developed within the field of soft robotics that begins to address technical challenges faced when developing shape-changing displays. For example, modular origami robots have the potential to be used to generate reconfigurable surfaces.

Mori (Belke and Paik, 2017) consists of single entities in the shape of equilateral triangles that combined form a modular reconfigurable surface. These self-folding robotic systems support modularity, origami-folding, mobility, and versatility in the shape output possibilities that go beyond traditional Human-Computer Interaction (HCI) implementations. These modular origami style robots can be easily adopted for shape-changing displays. Micro-robots are already beginning to be applied as an alternative technical implementation for developing data physicalizations and shape-displays (Le Goc et al., 2019). Though current work within the HCI field also focuses on more of a technical approach for combining modular robotic components. Zooids (Le Goc et al., 2016) are custom-designed wheeled micro-robots each 2.6 cm in diameter that can create swarm-based interfaces. These examples of robotics adapted for interfaces show promising future direction within the field of HCI, however, no substantial work has yet been contacted on their usability with users.

Flexible fabric actuators (Funabora, 2018) are also an emerging alternative for developing deformable surfaces without cumbersome electronics. These fabric actuators consist of lightweight and flexible artificial muscles that use electro-pneumatic regulators to create thin artificial muscles on a flexible rubber swath. The continuous surface system can control the fabric actuator smoothly, and control methods to realize six basic movements. An external depth camera can be used for supporting gestural user interaction capabilities with the actuated fabric surface. This particular hardware system has a lot of potential for adoption in the field of shape-changing displays due to its streamlined and thin nature.

### 3D Printing

Wong and Hernandez (2012) review current additive manufacturing processes for 3D printing. Our work focuses on Stereolithography (STL) and Fused Deposition Modeling (FDM) for 3D printing interlinked surfaces, as they are common and commercially available. As interest for 3D printing widens, marker communities such as MakerBot's (2018) and MyMiniFactory (Foresti et al., 2013) support users to share, collaborate, and further evolve new and pre-existing work. Recent research (Pei et al., 2015; Sabantina et al., 2015; Tenhunen et al., 2018) combines 3D printed polymers with textile materials to show new application opportunities, such as adaptive wearables. Users in maker communities have further developed these methods of 3D printing on fabrics to create flexible surfaces with more accessible methods (UncleJessy, 2018). 3D printing solid elements onto textiles offers opportunities to develop new materials that mimic fluid and ridged characteristics. However, uniform fabric lacks control designed interlinks provide.

3D printing interlinked cloth-like materials is an emerging applicating area (Rosenkrantz, 2016). Nervous System (2013), a design studio led by Jessica Rosenkrantz developed Kinematics (Rosenkrantz, 2013), a system for 4D printing that creates complex, foldable forms composed of articulated modules. The system provides a way to turn a three-dimensional shape into a flexible structure using 3D printing by modeling triangles and then interlinking the individual parts together with hinges. Our work reflects Kinematics' use of 3D printed articulated modules interlinked to construct a dynamic mechanical structure, but we apply this technique specifically for shape-changing display design and fabrication. Recent research (White et al., 2015) also shows electrospinning (Electroloom) as an approach for 3D printing custom 3D fabrics and textiles. As this technology remains in a prototyping phase, we focus on more accessible approaches for 3D printing fabrics using SLA and FDM machines. Our initial explorations are based on current design work for 3D printing fabric-like surfaces (Jeon, 2014; Montes, 2017), that can be easily accessible to researchers and designers.

## Fabrication Approach

This chapter presents an overview of the fabrication approach that demonstrates: (1) 3D printing complete and partial segments of interlinked surfaces with no additional support structures to reduce material consumption; (2) continuous and curving 3D printed interlinked surfaces; (3) with a reduced number of actuators that still create complex surface deformations; (4) using horizontal force to render tunnels and 2.5D cylindrical/oval forms; (5) under the surface projection as a form of visualization; and (6) embedding conductive materials as part of the surface for capacitive touch sensing.

The core premise of the fabrication approach is to use continuous 3D-printed surfaces, comprising of panels that are interlinked (Figure 1A), to create shape-changing surfaces that can be actuated with horizontal force. The following subsections explore design parameters to establish the utility of this fabrication approach. Scaling factors were tested to find the most error-free 3D printing approach. Actuation explorations established that horizontal force can be used to achieve a range of surface deformations and elevations. Visualization explorations adopted under-the-surface projection to reduce occlusion and embedded interaction capabilities reduced the need for external depth cameras for touch detection on the surface.

### Surface Scaling Based on 3D Printing Approaches

**Figure 2:**
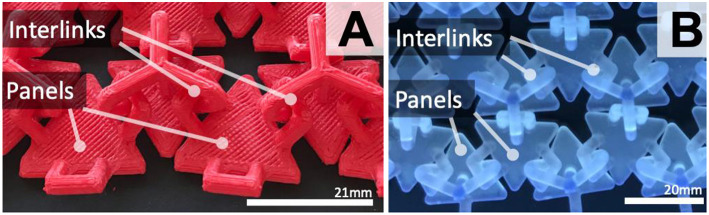
Bottom side of the surfaces. Interlinked triangular panels 3D printed (FDM) with red filament—Panel 21 × 19 mm and interlink width 4 mm **(A)**; SLA with clear resin—Panel 20 × 17 mm and interlink width 3 mm **(B)**. 3D model source.

To establish which additive manufacturing techniques produce the fewest print errors and highest resolution, scaling CAD parameters were explored. Stereolithography (SLA) 3D printing, using liquid resin (print resolution = 0.05 mm) achieved fewest errors with smaller scale factors. Clear resin also supports optical clarity for visualization opportunities with both projections and LEDs. To reduce material waste during fabrication, the surface was printed directly on the build plate with no support structures. Fused Deposition Modeling (FDM), was also tested (MakerBot Replicator2) to ensure the approach can be generalized. Figure 2A shows an FDM test surface (print resolution = 0.2 mm). In comparison to the SLA test surfaces (Figure 2B), dimensions of individual panels and interlinks using FDM are scaled up to ensure interlinks are strong enough for robust movement.

A multitude of panel shapes were tested during the initial surface design explorations including triangular (Figure 2) and square (**Figure 4A**). It is recommended that interlinks should be at least 3 mm width with FDM printing, as initial tests with smaller panels and interlinks resulted in increased print fails and inconsistencies. For larger scale surfaces, FDM could be used. Using clear or white filament/material supports projection. A greater number of panels and interlinks creates more detailed surface deformations and more fluid movement. Scale must be increased with FDM to ensure interlinks are properly formed without print faults. With SLA, interlink width of 2 mm for robustness is recommended.

### Actuation Explorations

Our work aimed to explore an alternative actuation approach for surface deformations and elevations that go beyond traditional linear vertical pin-arrays. The goal was to use fewer actuators than current state-of-the-art (Taher et al., 2017b) whilst maintaining high shape-output deformations.

In initial tests, horizontal force was used for surface actuation as opposed to vertical force, commonly applied with pin-array shape displays. The actuator consisted of two continuous servos, and two Micro-Bits (Micro:bit Micro:bit Educational Foundation., 2018) (one for servo control, one for user input). For early-stage testing, we explored the effects of continuous horizontal motion on surface deformation without fixed actuators. The test surface dimensions are 185 × 150 × 17 mm. Each triangular panel was 14 × 12 × 2 mm with interlink width of 2 mm.

**Figure 3:**
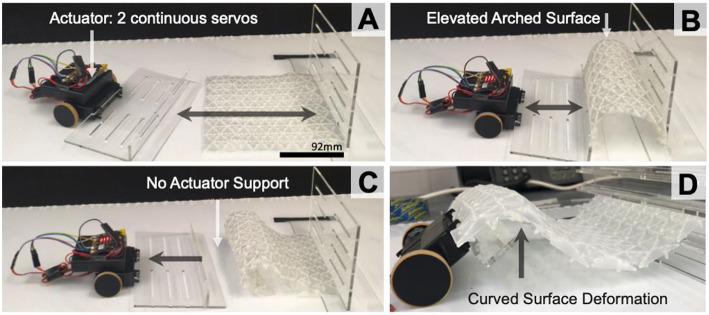
Horizontal uniformed force on 1 side of the surface **(A)**; for an elevated arch **(B)**; Surface deformation without actuator support **(C)**; and curved when the surface is slightly raised **(D)**.

A hexagonal design, with alternate linkages, was also tested (Jeon, 2014). It generated a uniform arch using the whole surface. Four forms of surface deformations and movements were achieved with horizontal force actuation. (1) Figures 3A,B shows continuous elevated movement from a flat surface to a high arc. (2) Once the actuator is paused, the surface stays in place without continuous force applied by the actuator. (3) When curving one side of the surface under itself the surface retains ridged form without any support required from the actuator (Figure 3C). (4) A wave shape form can be achieved when one side of the surface is higher (Figure 3D).

### Visualization Technique

**Figure 4:**
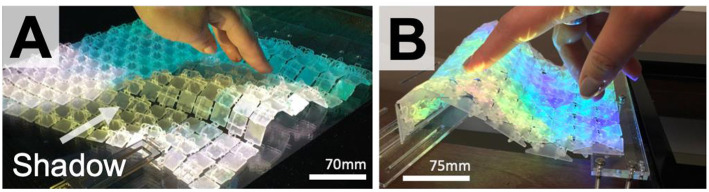
Visualization examples using over the surface **(A)** and under the surface **(B)** projection.

The visualization explorations aimed to reduce the issue of occlusion whilst maintain high-resolution visual output on the surface. Figure 4 shows two possible visualization approaches using a projector. Figure 4A shows over-the-surface projection suffering from occlusion; this is a common issue for shape-changing displays. Under the surface projection, using a table with a gap cut into it eliminated occlusion (Figure 4B). Though more space is required under the surface to situate the projector, no occlusion occurs when users interact with the display, creating a more impactful user experience.

As an alternative to projection for visualization, digital surface mount LEDs can also be embedded into each of the panels with minor adjustments to the 3D models (e.g., adding small gaps in the panels for situating the surface mount LEDs) before 3D printing. Though this is a lower-resolution alternative and would require additional circuitry and wiring, this visualization approach does eliminate the need for external components (e.g., projectors) to create a singular integrated deformable surface.

### Embedding Interaction

**Figure 5:**
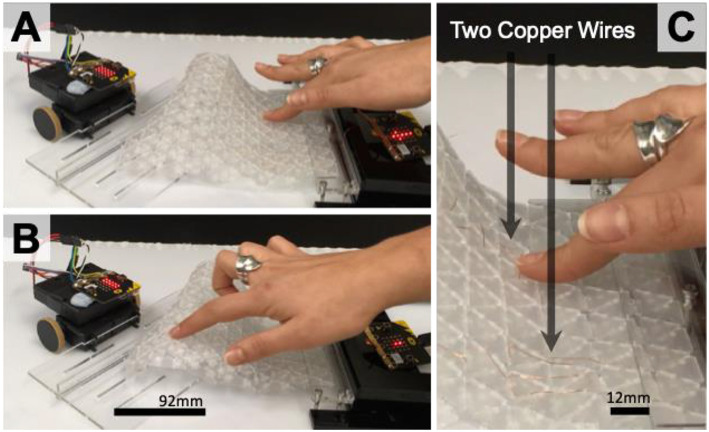
Interaction for controlling actuation embedded **(A,B)** using two fine copper wires interwoven through the surface for capacitive sensing **(C)**.

Exploring opportunities for embedded interaction capabilities within a surface aimed to reduce the need for external depth cameras for touch detection. Figure 5 demonstrates how capacitive touch can be embedded into the interlinked surface for controlling actuation. Two 0.1 mm copper wires were interwoven through the surface and connected to a 2nd MicroBit for capacitive touch sensing (Figure 5C). When touch is detected, the 2nd MicroBit will send a Bluetooth signal to the 1st MicroBit controlled robot to move and deform the surface. This approach enables the continuous surface to actively deform under the finger. Though the MicroBit originally only supports resistance detection, we were able to implement a conversion algorithm (Byford, 2018) that enables capacitive style sensor capabilities for prolonged interaction with the deformable surface. Though a novel interaction experience, accurate control of the surface movements was limited due to noise. As an alternative approach, an Arduino based capacitive sensing implementation can also be used with the CapSensing library (Badger, 2018). This approach follows a similar setup but also requires a medium to high value (100 kilohm −50 megohm) resistor attached to the copper wire.

In addition to copper wires, Conductive Silver Ink and ITO (Indium Tin Oxide) coated film can also be used for capacitive touch sensing on the surface, as a second layer of material. The main issue with using ITO film is that it may fracture and stop working during extensive surface deformations. From an additive manufacturing perspective, FDM (Fused Deposition Modeling) multi-material 3D printing can be used with conductive filament to print capacitive sensors directly into the surface.

### Surface Design Explorations

A range of geometries were explored to understand how the shape of each link and place can affect the movement and deformations of the surface as a whole. This is key for establishing what kinds of shape-output the surface can achieve during reconfigurations. The impact of varying panel and interlink (Figure 1) dimensions that influence surface motion and rigidity was also explored as part of this work. Fusion360 motion studies informed design choices for optimal interlink and panel design for fluid movement.

#### Panel Design

**Figure 6:**
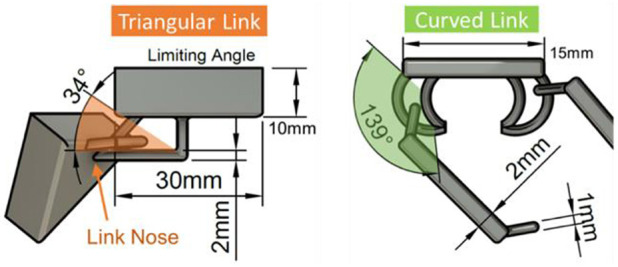
Interlink CAD design (triangular) with a limited angle for restricted movement on a triangular panel; and interlink design (curved) for more movement on a square panel.

Figures 6, **8** show interlinks and panels. Thinner panels (<3 mm) with rounded edges allow more fluid (e.g., smoother and unhindered) movement during elevation and horizontal deformations. This is because each of the plates in aggregate creates uniformed movement. Downscaling interlink width (≤1 mm) provides less under-the-surface protrusion but increases fragility. To overcome this, resin that simulates ABS (Acrylonitrile Butadiene Styrene) injection molded components is used for tougher material properties to mitigate fragility with thinner interlinks. However, the blue tint of the resin decreased optical clarity for visualization. Thicker panels (>3 mm) with smaller spacing between interlinks (**Figure 8**) provide rigidity and robust support when the surface is deformed.

However, scaling up panel dimensions in the Y-axis results in courser geometry and limited movement flow, especially when interlinks are tightly coupled. Triangular, square, and hexagonal panels were designed and fabricated to understand how panel shape can affect surface deformations. Size of panels and interlinks has a greater impact on surface movement, as these parameters affect individual plate rotation and movement.

#### Interlink Motion Explorations

Motion studies were performed on two initial interlink designs. A planar joint was used to test freedom of movement with each interlink design. Constraints were set to ensure only motion inside the interlink was rendered. Reduced space within the link, see Figure 6 (approx. ≤1 mm) limits the movement. Too much space within interlinks (≥3 mm) creates very lose panel movement, resulting in loss of fluidity in motion and the continuous surface shape. As seen in Figure 6, triangular links have a much more limited angle of movement (34°) in comparison to curved links (139°). Approximately 2 mm space for interlinks gaps (see Figure 6 green and orange shaded areas) is recommended to ensure panels create fluid motion but are not too loose.

The triangular interlink design (Figure 6 left), shows that the angle for movement is limited to 34° due to the nose of the interlink (Figure 6 left). This type of interlink could be used in specific areas of a display to create more ridged deformations. A curved interlink (Figure 6 right) provides a 139° angle for panel motion. Curved interlinks allow a set of panels to drape, like cloth, whereas a triangular interlinks support rigidity and self-support for surface deformations. Self-support for triangular shaped links occurs due to the link nose limiting the bending of the connected link (Figure 6 left) and in aggregate this effect is propagated to create a self-supporting surface.

#### Horizontal Actuation and Shape-Output Control

**Figure 7:**
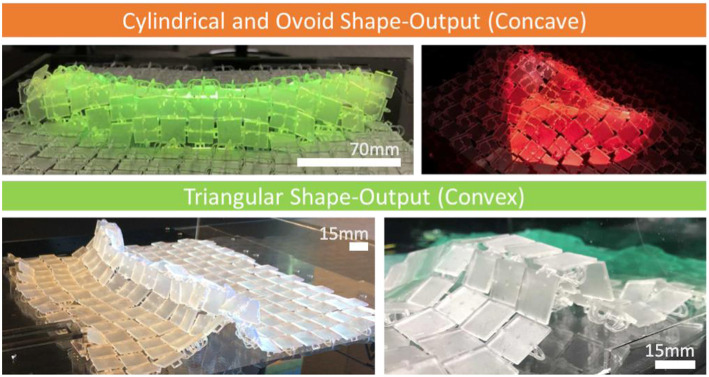
Examples of cylindrical and ovoid shape-output when links are on top and convex shape-output when links face down.

For larger scale display surfaces we used ShapeClips (Hardy et al., 2015) as modular linear actuation mechanisms. These modular actuators can be re-configured to suit a range of morphologies and follow a “plug and play” set-up. For cylindrical/ovoid and triangular shape-output (Figure 7) accuracy and control, speed and force of actuation are key factors. To control shape position, the more force and speed propagated through the surface, the further away surface elevation occurs from the actuator. To control shape-output scale, a greater “push” area of an actuator increases the width of the shape. Figure 9A shows a cylindrical shape with one actuator. When two actuators increase the “push area” (Figure 9B), with the same force at the same speed, the shape-output width is increased across the surface.

Each side of the surface has specific shape output characteristics based on the freedom of the angle of movement. To render oval/ovoid and curved 3D forms, the surface needs to have the links facing up (see Figures 7, **9A,B**). As the angle of movement is restricted by adjacent panels' edges, the surface in aggregate bends in an oval fashion and can render tunnel oval like structures (Figures 7, **9B**). The curvature continuity of the surface when links are facing up enables physical 2.5D renderings of spheres, cones, and cylinders (Figure 7). To physically render 3D shapes with sharper corners and edges it is best to have the surface links facing down as this creates a more “pointed” shape elevation (Figures 7, **9C**).

Each side of the surface has specific shape output characteristics based on the freedom of the angle of movement. To render oval/ovoid and curved 3D forms, the surface needs to have the links facing up (see Figures 7, **9A,B**). As the angle of movement is restricted by adjacent panels' edges, the surface in aggregate bends in an oval fashion and can render tunnel oval like structures (Figures 7, **9B**). The curvature continuity of the surface when links are facing up enables physical 2.5D renderings of spheres, cones, and cylinders (Figure 7). To physically render 3D shapes with sharper corners and edges it is best to have the surface links facing down as this creates a more “pointed” shape elevation (Figures 7, **9C**).

Having the surface positioned where the links are facing down, enables more freedom in the angle of movement between each panel. As a result, the panels in aggregate can be bent to much greater angles without the limit of touching the other panel edges. When the surface links are facing down (Figure 7) shapes such as triangular pyramids, square based pyramids, and triangular prisms can be rendered. To achieve these shape-outputs using horizontal actuation, the actuators need to be driven at different speeds and force.

## Surface Applications

**Figure 8:**
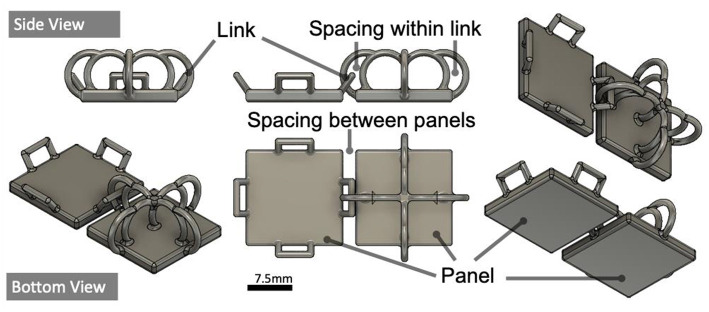
Optimal panel (15 × 15 × 2 mm) & interlink (width = 1 mm) designed, with space between panels = 2 mm.

The proof-of-concept surface combines under the surface projection for visualizations and linear motors for horizontal actuation in two applications. Figure 8 shows the square panel and interlink design chosen for fabricating a larger 280 × 280 mm display surface. We reduced the interlink width to 1 mm. Though this allows for finer aesthetic, the surface becomes more fragile, prone to breaks and fractures. An interlink width of 2 mm is optimal for a robust surface that can withstand excess force and deformations.

**Figure 9:**
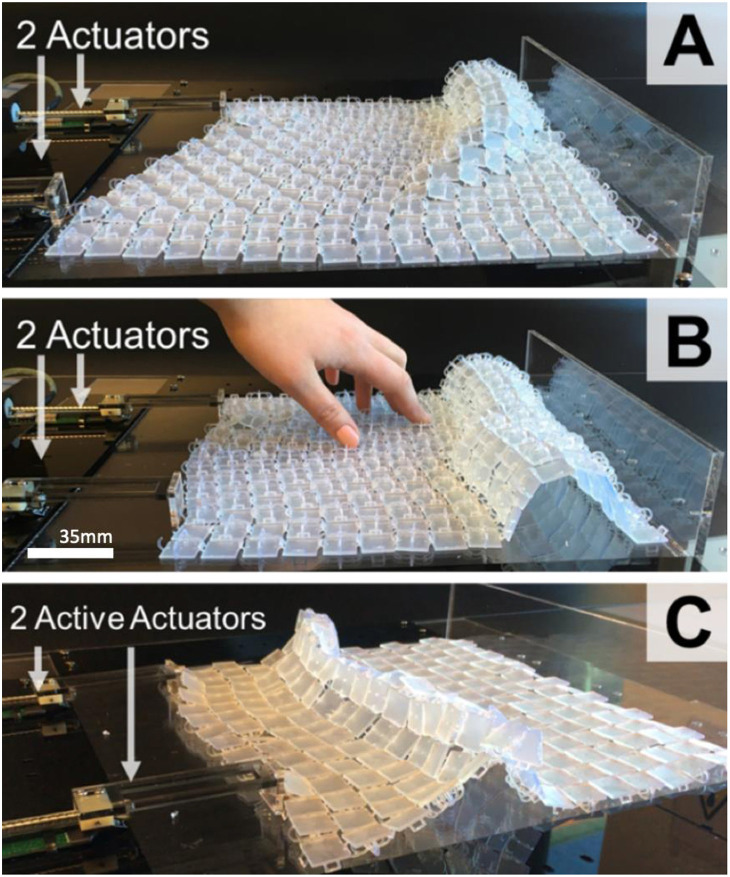
Surface (280 × 280 × 8 mm) with 2 actuators attached to 1 side. Interlinks on top with shape-output only on the far side **(A)**, increased width of cylindrical shape when two actuators push areas used **(B)**, and flat panels on top of the surface for “pointed” shape elevation **(C)**.

Due to the limited build platform space on the Form2 (145 × 145 mm), four interlinked surfaces were 3D printed separately (140 × 140 × 8 mm) and “welded” together, using a glue gun, to create a larger surface (280 × 280 × 8 mm), see Figure 9. Each surface consisted of two panel/interlink designs, seen in Figure 8, iterated to create an 8 × 8 grid (140 × 140 × 8 mm).

Total print time for a 280 × 280 × 8 mm surface was 15, and 1 h 20 min for post-processing (20 min per print). Both sides of the surface have specific characteristic deformations. Sharper surface forms are rendered when interlinks of the surface face down (see Figure 9C), as each panel has a greater angle of movement. When interlinks are facing up, a curved form is elevated (Figure 9A) due to the limited angle of movement for each panel.

### Surface Applied to Existing State-of-the-Art

**Figure 10:**
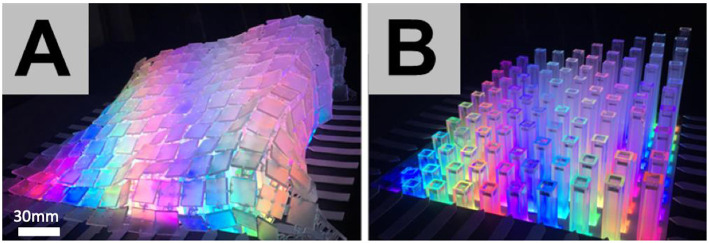
Interlinked surface over linear pin-array **(A,B)**.

To demonstrate generalizability with existing technologies, the surface was used to transform large scale vertically-actuated pin-arrays into continuous surface shape-changing displays. EMERGE (Taher et al., 2017a), a 10 × 10 array of actuated pins, was selected for this as it supports under-the-surface visualization. Figure 10 shows that the surface creates a continuous display. When actuators are spread further apart the surface renders continuous shape-forms. Translucent panels release light from LEDs in each pin actuator to create diffused visualization. The surface required no attachments to pins and rendered an organic fluid movement during actuation, which could better represent continuous mathematical functions or topography without the need for a cloth layer.

### 3D Printed Surface as a Stand-Alone Display

**Figure 11:**
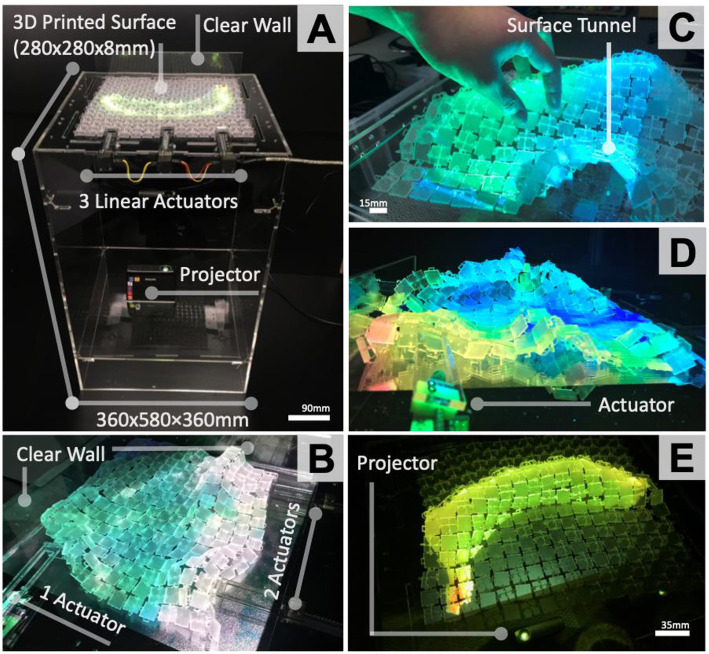
Shape-Changing display set-up with under-the-surface projection to eliminate occlusion, 3 actuators on one side of display **(A)**; Wave simulation application with 3 linear actuators **(B)**; User manipulating surface with a tunnel **(C)**; Temperature simulator for reef topography **(D)**; Surface rending 2.5D cylindrical form—banana **(E)**.

Figure 11A shows a shape-changing display with rear-projection that uses three actuators. A layer of clear laser-cut Perspex is used to secure actuators on the sides and also ensures the fabric-like surface does not droop. The use of horizontal force as an actuator eliminates the need for electronics under the surface and also deforms in both the X and Y axis, as seen in Figure 11B. The display also renders under-the-surface “tunnels” (Figure 11C) whilst a laser-cut clear “wall” is used on one side of the display to ensure the surface elevates when an actuator pushes it.

### 2.5D Oval and Cylindrical Object Rendering

The surface was first used with the links facing up to physically render cylindrical and oval forms. When designing possible content for this first shape-changing display, multiple examples of cylindrical and oval shape-forms were considered for rendering in 2.5D.

Based on insights from the content generation studies (Sturdee et al., 2015), physically showing the scale of various food items was selected as an application scenario to explore. The design focus of this initial shape-changing display was to demonstrate to users the physical scale of food items at a restaurant (e.g., pizza size or banana). Figure 11E shows an example of a 2.5D banana form with rear-projection for imagery. Users could physically see the size of certain food at a restaurant before they order it. Two actuators, on one side of the display, elevated areas of the surface as seen in Figure 9. A user can further refine the oval and cylindrical shape-outputs by controlling the distance an actuator pushes the surface backwards or forwards, or by manipulating the surface deformations by hand as seen in Figure 11C. This set-up could also be used in an architectural context to render tunnels.

### Physical Flow Simulations

Figures 11B,D show the surface as a display to simulate “flowing” visualizations with physical shape-output. A physical wave motion simulation (Figure 11B) was used as an example to show natural flowing movement throughout the continuous surface. Two linear actuators were used on a single side of the surface and another one on the perpendicular side.

The actuators act as mechanical paddles that move back and forth either simultaneously or individually to create different types of wave scenarios based on horizontal actuation speed and force. Figure 1C shows a close-up of surface deformation during the actuation for simulating wave shaped forms. Figure 11D shows the topography of a reef that gradually changes shape as the visualization, and water temperature varies.

## Discussion

We present initial explorations of 3D printed interlinked panels to fabricate dynamic surfaces for shape-changing displays. These surfaces can be scaled by combining multiple prints as a “patchwork” to create larger surfaces. Fluidity of continuous surface movement with added rigidity enables cylindrical, oval, and tunnel shape-forms. Clear resin, used during fabrication, enables visualizations with no occlusion. To demonstrate actuation opportunities we used horizontal force, with a reduced number of actuators, for surface deformations in both X and Y-axis.

**Figure 12:**
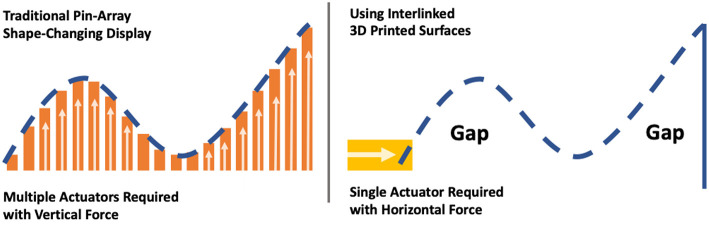
Comparison of actuators required using traditional pin-array shape-displays **(Left)** and using an interlinked 3D printed surfaces to achieve the same deformation with horizontal actuation force **(Right)**.

The initial explorations into actuation opportunities highlight the use of horizontal force to achieve shape deformations without the need for linear actuators to be positioned below the surface. As seen in Figure 12, using horizontal force can provide the same curvature of surface deformation as a traditional pin-array display (Figure 12—Left), but with a significantly reduced number of actuators (Figure 12—Right).

Unlike with traditional pin-array shape-changing displays, which use vertical linear force, having the linear actuators positioned on the sides of the deformable surfaces also allows for additional opportunities for visualization, such as under-the-surface projection. By reducing the area needed to be covered for shape deformations, fewer actuators are needed to be positioned on the outside edges of the display in comparison to uniform pin-arrays that are currently used. However, the level of control required for shape deformations is limited with horizontal force for actuation.

The granularity of shape-out, defined by Kim et al. (2018) as the density of physical actuation points, is limited with horizontal force as the actuation in the initial exploration conducted for this work is focused on uniformed force that is applied to one whole side of a 3D printed surface. With the larger example of the shape-changing display prototypes developed (Figure 11), three actuators are positioned to apply horizontal force on more specific areas of the surface edge. Based on the surfaces' layout, it can demonstrate retained fluidity and rigidness whilst rendering cylindrical, oval, and tunnel forms as seen in Figure 11. Though granularity is increased with the number of physically actuated points on the surface, the level of control for actuating each specific point on the surface is still not accurate in terms of modeling precise deformation and elevation. This especially applies to areas at the center of the display, where the propagated horizontal force is not as focused.

As mentioned earlier, there is a trade-off between shape resolution and number of actuators. This is a scaling matter, for both surface dimensions and actuation mechanism used. A larger surface requires more actuators to move different areas of the surface. The accuracy of shape elevation when using linear force is determined by the actuator's capabilities to: (1) control its speed and force applied to the surface, (2) the “push” area of the actuator, and (3) its actuated extension length. To increase the number of oval/cylindrical shapes rendered across a larger display requires the actuators to be more spread across the edge of the surface.

Regarding scaling of the individual panels, both size and morphology are adaptable as desired. The fabrication dimensions are not limited to the implemented 15 × 15 × 2 mm sizing but can be further reduced. Panel dimensions can be downscaled to 5 × 5 × 2 mm if required for higher resolution. When downscaling, the spacing between each of the panels needs to be taken into consideration. Particularly, as spacing between panels has the most effect on the deformations achieved by the surface in aggregate. We recommend the minimum spacing of 1 mm between each of the panels for link joints, as anything smaller than 1 mm spacing will cause the surface to become ridged as a whole where links are fused together during printing. Additionally, though we predominantly used a square panel design for our prototypes, triangular and hexagonal panel designs were also explored to observe the effects of morphologies on the general deformations of the surface in aggregate. When comparing the deformations achieved by the triangular and hexagonal panel designs, the number of panel sides had little effect on the overall deformations achieved in aggregate. Rather, the fluidity and deformations possible were affected by the spacing of the links between the panels rather than the shape of the panels.

As an additional scaling factor, the overall surface dimensions are also affected by horizontal actuation. Ultimately, when horizontal force pushes the edges of the surface to achieve elevation, the surface shrinks on the X-axis and the edges are pushed inwards. To mitigate the shrinking screen effect with horizontal actuation, future implementations of the surface will have extended areas where the edges of the screen can be rolled up and hidden until actuation occurs where they can expand as required.

In future work, we also aim to further support opportunities for fabricating dynamic shape-changing displays by integrating actuation, visualization, and interaction within the display surface. By interweaving fine 0.1 mm copper wire throughout the surface we begin to embed electric components for interaction. 3D printing with conductive material and embedding LEDs into the surface panels will further support electronic component integration within the surface. Our goal is to design and fabricate a range of robust dynamic shape-changing displays that are deployed in a range of contexts, particularly in public environments. This will support formal quantitative and qualitative user evaluations to understand user engagement with shape-changing displays.

From an application background for deformable surfaces within the context of shape-changing displays, our demos are based on current use-case examples (Sturdee et al., 2015). For future implementations, we hope to adopt our deformable surfaces based on applications for various purposes (Everitt et al., 2016), such as physical terrain mapping (Everitt and Alexander, 2017). The current application background for these deformable surfaces comes from the area of tangibles and smart matter (Ishii et al., 2012) and our future applications will hope to explore new forms of dynamic data physicalizations.

## Conclusion

This work presents an exploration of 3D printed surfaces as a fabrication technique for shape-changing displays. We described our general fabrication approach that demonstrates opportunities for under-the-surface visualization and embedding interactive components into the surface. By varying surface design parameters, we can retain fluidity and rigidness whilst rendering cylindrical, oval, and tunnel forms with a reduced number of actuators, and horizontal force actuation. We also show two possible applications of the surface based on the current shape-out possible with our initial surface design. We believe this fabrication technique will further enhance the design and development of shape-changing display by supporting dynamic deformations through a balance of ridged and fluid material characteristics.

## Data Availability

All datasets generated for this study are included in the manuscript/Supplementary Files.

## Author Contributions

All authors listed have made a substantial, direct and intellectual contribution to the work, and approved it for publication.

### Conflict of Interest Statement

The authors declare that the research was conducted in the absence of any commercial or financial relationships that could be construed as a potential conflict of interest.

## Supplementary Material

Supplementary Video 1 Supplementary material includes a video demonstrating horizontal actuation explorations and embedded interaction capabilities supported by the 3D printed deformable surface, with a final application demo example.
